# A novel chronic dural port platform for continuous collection of cerebrospinal fluid and intrathecal drug delivery in free-moving mice

**DOI:** 10.1186/s12987-022-00331-1

**Published:** 2022-05-03

**Authors:** Tsuneo Nakajima, Shuko Takeda, Yuki Ito, Akane Oyama, Yoichi Takami, Yasushi Takeya, Koichi Yamamoto, Ken Sugimoto, Hideo Shimizu, Munehisa Shimamura, Hiromi Rakugi, Ryuichi Morishita

**Affiliations:** 1 Department of Geriatric and General Medicine, Graduate School of Medicine, Osaka University, Suita, Osaka 565-0871 Japan; 2 Department of Clinical Gene Therapy, Graduate School of Medicine, Osaka University, Suita, Osaka 565-0871 Japan; 3 Department of Internal Medicine, Osaka Dental University, Hirakata, Osaka 573-1121 Japan; 4 Department of Neurology, Department of Health Development and Medicine, Osaka University, Suita, Osaka 565-0871 Japan; 5 Osaka Psychiatric Research Center, Osaka Psychiatric Medical Center, Hirakata, Osaka 573- 0022 Japan; 6 Department of Clinical Nursing Division of Health Sciences Graduate School of Medicine, Osaka University, Suita, Osaka 565-0871 Japan; 7 General and Geriatric Medicine, Kawasaki Medical School General Medical Center, Okayama, 700-8505 Japan

**Keywords:** Cerebrospinal fluid, Biomarker, Mouse model, Central nervous system disorder, Intrathecal space, Real-time monitoring

## Abstract

**Background:**

Cerebrospinal fluid (CSF) provides a close representation of pathophysiological changes occurring in the central nervous system (CNS); therefore, it has been employed in pathogenesis research and biomarker development for CNS disorders. CSF obtained from valid mouse models relevant to CNS disorders can be an important resource for successful biomarker and drug development. However, the limited volume of CSF that can be collected from tiny intrathecal spaces and the technical difficulties involved in CSF sampling has been a bottleneck that has hindered the detailed analysis of CSF in mouse models.

**Methods:**

We developed a novel chronic dural port (CDP) method without cannulation for CSF collection of mice. This method enables easy and repeated access to the intrathecal space in a free-moving, unanesthetized mouse, thereby enabling continuous long-term CSF collection with minimal tissue damage and providing a large volume of high-quality CSF from a single mouse. When combined with chemical biosensors, the CDP method allows for real-time monitoring of the dynamic changes in neurochemicals in the CSF at a one-second temporal resolution in free-moving mice. Moreover, the CDP can serve as a direct access point for the intrathecal injection of CSF tracers and drugs.

**Results:**

We established a CDP implantation and continuous CSF collection protocol. The CSF collected using CDP was not contaminated with blood and maintained physiological concentrations of basic electrolytes and proteins. The CDP method did not affect mouse’s physiological behavior or induce tissue damage, thereby enabling a stable CSF collection for up to four weeks. The spatio-temporal distribution of CSF tracers delivered using CDP revealed that CSF metabolism in different brain areas is dynamic. The direct intrathecal delivery of centrally acting drugs using CDP enabled real-time behavioral assessments in free-moving mice.

**Conclusions:**

The CDP method enables the collection of a large volume of high-quality CSF and direct intrathecal drug administration with real-time behavioral assessment in free-moving mice. Combined with animal models relevant to CNS disorders, this method provides a unique and valuable platform for biomarker and therapeutic drug research.

**Supplementary information:**

The online version contains supplementary material available at 10.1186/s12987-022-00331-1.

## Background

Cerebrospinal fluid (CSF) provides a close representation of the pathophysiological changes occurring in the central nervous system (CNS) and has therefore been used for pathogenesis research and biomarker development for CNS disorders [1, 2]. CSF biomarkers have proven useful for the diagnosis and prognosis of many neurodegenerative disorders [3–5]. However, the molecular mechanisms of most CNS disorders remain largely unknown; therefore, the use of mouse models combined with the analysis of CSF markers is required to facilitate research.

The mouse is the most commonly used model organism for human disorders and has served as a useful and adaptable tool for researchers performing basic and preclinical research [6]. Numerous mouse models relevant to CNS disorders are available and have played a significant role in exploring the molecular mechanisms underlying the disorders, testing the efficacy of candidate drugs, and developing CNS biomarkers [7, 8]. The CSF obtained from valid mouse models relevant to CNS disorders can be an important resource for successful biomarker research; however, the ability to collect only a limited volume of CSF from a mouse and the technical difficulties related to CSF collection procedures have been bottlenecks that have hindered the detailed analysis and comprehensive profiling of metabolites and proteins in the CSF [9].

Three basic methods have been reported and used for mouse CSF collection [9, 10]. The most commonly used technique is a single collection by cisterna magna (CM) puncture, in which CSF is withdrawn using a pipette or fine glass capillary via a small hole made in the membrane covering the CM [9–11]. This method has been widely used for mouse CSF research; however, the amount of CSF obtained is relatively small (~ 10 µL per mouse), and the procedure requires anesthesia and head fixation, which prevents CSF sampling under physiological conditions and could affect the quality and quantity of the collected CSF. Repeated serial collection from the same mouse is also difficult with this method. Another method is intrathecal cannulation, which involves inserting a fine cannula into the intrathecal space [12]. This method allows for repeated collection at different time points from the same mouse; however, its main drawback is tissue injury due to cannula insertion into the mouse’s narrow intrathecal space [13, 14]. The use of this method can be especially problematic when the collected CSF is intended for use in biomarker research targeting neurodegenerative marker proteins because markers, such as tau and neurofilament, could be artificially increased by contamination from damaged tissues [10]. A third option is CSF collection from the lateral ventricle via a needle stereotaxically targeting the ventricle [15]. This method can be used for CSF collection and drug administration directly into the brain; however, it requires that the needle penetrates the brain parenchyma, thereby increasing the susceptibility to an artifactual increase in neurodegenerative markers [10].

The ideal method for collecting CSF from mice has numerous specific requirements, as it must allow for (1) the collection of a large volume of CSF from a single mouse, (2) a long-term continuous collection from the same animal, (3) CSF collection under physiological conditions in an awake, free-moving mouse, and (4) repeated serial collections at different time points. This ideal CSF collection method would enable a detailed comprehensive profiling of CSF components, monitoring of longitudinal changes in biomarker levels, and determining the effects of centrally acting drugs. The choice of method is also important from an animal ethics viewpoint. Therefore, the ideal method should also reduce the number of mice needed for CSF experiments.

The methods adopted for CSF collection can also be used for drug or tracer administration into the intrathecal space [12]. The penetration of most drugs is restricted by the blood–brain barrier; therefore, many small drug compounds and proteins cannot enter the CNS [16]. Moreover, the blood–CSF barrier, formed by choroid plexus epithelial cells, limits drug entry into the CNS [1, 2]. Intrathecal administration therefore has advantages over other drug delivery procedures, such as oral and intravenous administration, given that it reduces side effects in the peripheral tissues and provides direct and rapid exposure of the CNS to the drug. Intrathecal delivery has been employed for certain CNS disorders in clinical settings [12], and its use has been reported for administering drugs to mice; however, this method poses a risk of tissue injury due to cannula insertion [12]. Therefore, developing a method for stable and repeated intrathecal administration with minimal risk of tissue damage would be useful for developing therapies and for evaluating the dynamic metabolism of CNS tracers.

In this study, we describe the development of a chronic dural port (CDP) method as a novel approach for mouse CSF collection and intrathecal drug delivery. The CDP is installed on the CM along with CSF collection tubing fixed using dental cement. This system allows for easy and repeated access to the CSF, and the connection to a roller/syringe pump enables continuous long-term CSF collection from a free-moving, unanesthetized mouse. This method enables the collection of a large volume of high-quality CSF from a single animal. The CDP can also serve as a direct access point to the intrathecal space, thereby allowing direct intrathecal drug administration and real-time behavioral assessment in free-moving mice.

## Methods

### Animals

All animal experiments were performed in compliance with the Guidelines for the Care and Use of Laboratory Animals at Osaka University School of Medicine and the National Institutes of Health Guidelines for the Care and Use of Laboratory Animals. Three–four-month-old male mice (C57BL6/J) weighing 20–25 g were used in the study (obtained from CLEA Japan). Seven-month-old male PS19 tau-transgenic mice expressing human P301S 1N4R mutated tau driven by the PrP promoter (The Jackson Laboratory) [17] and wilt-type littermates were also used for measuring CSF tau. The animals were maintained at room temperature (25 °C ± 2 °C) under a standard 12-h/12-h light-dark cycle, with free access to water and food.

### Surgical procedure for CDP implantation

Polyurethane CSF collection tubing for CDP (0.3 mm i.d.; M025V-100, Eicom, Japan) was cut to a length of 3 cm using a razor blade. The CSF collection tubing was connected to the fluorinated ethylene propylene (FEP) tube with a joint (JF-10, #800,160, Eicom, Japan), which was connected to a peristaltic pump (ERP-10, #600,100, Eicom, Japan) or microsyringe pump (ESP-64, Eicom, Japan). The tubing was filled with distilled water before surgery.

The mice were anesthetized with an intraperitoneal injection of a combination of domitor (0.75 mg/kg), midazolam (4 mg/kg), and butorphanol (5 mg/kg) and then mounted on a stereotaxic frame (SR-5 M-HT, NARISHIGE, Japan), with a heating pad placed under the body. The mice were positioned so that the head formed an approximately 80° angle with the body, secured by using the head adaptor. The surgical area was disinfected with iodine and covered with a surgical drape. Using straight-tip surgical scissors, an incision (20 mm) was made in the skin at the center of the neck. Under the dissection microscope, the subcutaneous tissue and muscle layers were separated by blunt dissection with forceps and fine-tipped cotton swabs to expose the atlanto-occipital membrane (AOM) of the CM.

A small hole was made in the center of the AOM by shallow centesis using a 30G needle, taking care not to damage the brain tissue and blood vessels. After confirming the CSF flowing out of the puncture due to cerebrospinal pressure, the tip of the CSF collection tubing was attached to the membrane by folding the tubing with a stereotactic arm so that it covered the small hole made on the AOM. The CSF was then withdrawn via a peristaltic pump at a flow rate of 20 µL/h, which visually confirmed that the CSF was being drained into the cannula. After confirming this, the flow rate was decreased to 10 µL/h.

The CSF collection tubing, AOM, and surrounding bone structures, including the occipital crest and anterosuperior border of the atlas, were fixed with 4-META/MMA-TBB resin (Super-Bond C&B, Sun Medical, Japan). After confirming that the CSF continued to be drained and that the cement was completely secured, the flow rate was further decreased to 4 µL/h.

A steel wire anchor was then attached to the skull, which tethered the mice with the sensor-integrated balance arm. The periosteum on the surface of the skull was removed using a swab. The wire anchor has a small loop that is fixed to the surface of the skull using the dental cement, which should be allowed approximately 10 min to harden before finally suturing the scalp.

The CSF collection tubing was disconnected from the FEP tube, and the outlet was plugged. The skin was aligned and sutured using 7 − 0 silk, and then a mixture of analgesic, sugar, and antibacterial agents was administered intraperitoneally. The mice were left on the top of the heating pad to maintain their body temperature until waking. The mice were then carefully transferred to a recovery home cage and were allowed at least four days for tissue recovery before starting the continuous CSF collection.

### Continuous CSF collection via CDP

Four days after the CDP implantation, the mice were tethered with the sensor-integrated balance arm and placed in the movement-response caging system (MD-1409, BASi, USA) as previously described [18]. The sensor detects the animal’s rotation and turns the cage in the opposite direction, allowing unrestricted movement by the animals without applying pressure to the probe assembly. The CSF collection tubing (M025V-100, Eicom, Japan) from the CDP was connected to a roller pump (ERP-10, Eicom, Japan) or syringe pump (ESP-32, Eicom, Japan) to withdraw CSF at a constant flow rate. Maintaining the constant flow of CSF prevents healing or closing the AOM and enables long-term collection (e.g., four weeks). The movement-response caging system was placed in the sound-attenuating box (Natsume Seisakusho, Osaka, Japan) to control the environmental conditions. The mice were maintained at room temperature (25 °C ± 2 °C) under a standard 12-h/12-h light-dark cycle, with free access to water and food. The mice’s locomotor activity during the experiment was measured by an infrared light beam crossing system as described below.

### A single-collection technique for mouse CSF via CM punctures

A conventional single-collection technique for mouse CSF was performed as previously described, with minor modifications [10]. Mice were anesthetized with an intraperitoneal injection of a combination of domitor (0.75 mg/kg), midazolam (4 mg/kg), and butorphanol (5 mg/kg) and then placed on a warm surgical platform. Under the dissection microscope, the subcutaneous tissue and muscles were separated to expose the CM. CSF was collected by puncturing the AOM with a 30G needle and aspirated with a P20 pipettor.

### Chronic CMc method

For the chronic CMc experiment, the implantation of a catheter into the CM was performed as previously described [12]. Three days after the CMc surgery, the mice were euthanized, and their brains were harvested for histology to evaluate the tissue damage due to the intrathecally inserted cannula.

### Assessment of blood contamination and measurement of CSF electrolytes

A high-sensitivity spectrophotometry method was employed to measure blood contamination in the collected CSF, as previously described [10]. Hemoglobin levels in the collected CSF, released using the hypotonic freeze-thaw method, were quantified by measuring absorbance at 417 nm on a NanoDrop ND-1000 spectrophotometer (NanoDrop Technologies/Thermo Scientific, Wilmington, DE, USA). We used clear CSF spiked with increasing amounts of whole blood (0.01%, 0.1%, and 1%) as references. Pooled CSF was centrifuged at 2000 g for 30 s to remove any red blood cells, and the supernatant was used as clear CSF. We also performed a direct blood cell count under a phase contrast microscope as a more sensitive measurement for detecting blood contamination. A 5-µL volume of CSF was loaded into a hemocytometer with a counting grid (Thoma Shoji Co. Ltd., Tokyo, Japan), and the number of red blood cells in four distinct areas measuring 0.0625 mm^2^ (0.25 mm × 0.25 mm) was counted. The mean number of cells for the four distinct areas was used for the analysis. The electrolyte concentrations (sodium, potassium, and chloride) in the collected CSF were measured by a blood gas analyzer (ABL700, Radiometer Medical, Denmark).

### Enzyme-linked immunosorbent assay measurements of ApoB, ApoE, and albumin

The concentrations of ApoB, ApoE, and albumin in the samples (single collection CSF, cCSF, and plasma) were determined by the mouse ApoB enzyme-linked immunosorbent assay (ELISA) kit (#ab230932, Abcam), the mouse ApoE ELISA kit (#ab215086, Abcam), and the mouse albumin ELISA kit (#ab207620, Abcam), respectively, according to the manufacturer’s instructions.

### Tau ELISA

The concentrations of total human tau in the single collection CSF and cCSF of tau-transgenic PC19 and wild-type mice were determined by Tau (Total) Human ELISA kit (#KHB0041, Invitrogen), according to the manufacturer’s instructions. Samples were diluted at 1:10 using a sample diluent buffer provided by the ELISA kit.

### Biosensor measurements of glucose and lactate in CSF

Enzyme-based biosensors were used for the real-time monitoring of glucose and lactate levels as previously described [19, 20]. In this study, the distal end of the glucose and lactate biosensor electrodes (Part #7004-80 - Glucose and #7004-80-Lactate; Pinnacle Technology, USA) were inserted into the biosensor port (Eicom, Japan), where the biosensors were exposed to the continuously collected CSF. All the biosensors were calibrated in vitro before implantation. Mice were administered a single dose of an intraperitoneal injection of glucose (2 g/kg body weight) during the biosensor measurements of CSF glucose and lactate.

### Brain histology

Brains were fixed in 4% paraformaldehyde for 24 h at 4 °C, incubated for 2 days in 30% sucrose in phosphate-buffered saline (PBS) and embedded in a Tissue-Tek O.C.T. Compound (Sakura Finetek Japan Co., Ltd.); 8-µm thick sagittal and coronal sections were then cut on a cryostat and mounted on glass slides. The brain sections were then stained with hematoxylin and eosin (H&E) to evaluate the tissue integrity and damage due to CSF collection. For immunostaining, the sections were permeabilized with 0.5% Triton X-100 in PBS, blocked in 5% bovine serum albumin in PBS, and immunostained with rabbit anti-glial fibrillary acidic protein (GFAP) antibody (1:200, PRB-571 C, BioLegend) and mouse anti-NeuN antibody (1:200, MAB377, Merck) in 1% bovine serum albumin in PBS at room temperature overnight. Sections were washed thoroughly in PBS; the immunoreactions were then visualized by fluorescent secondary antibodies. The sections were then mounted using VECTASHIELD medium (H-1000, Vector Laboratories). We used the following secondary antibodies: Alexa 488-conjugated goat anti-rabbit IgG antibody (1:200, ab150077, Abcam) and Alexa 594-conjugated goat anti-mouse IgG antibody (1:200, ab150116, Abcam). Fluorescent images were captured using a fluorescence microscope (BZ-9000, Keyence, Japan) equipped with a digital camera, using the same fluorescence settings in all cases. The pixel intensities of the fluorescent signal were analyzed and quantified using National Institutes of Health Image software. For the GFAP burden analyses, data are reported as the percentage of the labeled area captured (positive pixels) divided by the full area captured (total pixels).

### Drug and tracer injection via CDP

The intrathecal injection of the drug and the CSF tracer injection were performed using a 30G Hamilton microsyringe connected to the CDP via CSF collection tubing. The dead volume of the CSF collection tubing was approximately 0.6 µL. A single dose of contrast agent (5 µL in 10 s) was injected via the CDP, followed by sequential micro-CT imaging (R_mCT2, Rigaku Corp., Japan). We used an 821.14 molecular weight non-ionic, water-soluble radiographic contrast agent (iohexol, GE Healthcare, IL, USA). The concentrations of the contrast agents in each brain region at each time point were determined based on the signal intensity, which was used to calculate metabolic parameters, such as t1/2 and Tmax. For the histological assessment, a single dose of a water-soluble nuclear-staining dye (Hoechst 33,342, Invitrogen, USA) was injected via the CDP (50 µg/5 µL in 10 s). The brain was harvested at 60 min. Coronal sections were immunostained with an anti-NeuN antibody as described above, and fluorescent images were captured using a fluorescence microscope (BZ-9000; Keyence, Japan). For the behavioral assessment of an intrathecally delivered drug, an anesthetic agent (midazolam; Maruishi Pharmaceutical, Japan) was acutely injected via CDP in the free-moving mice (25 µg/5 µL in 1 s through a syringe connected to the CDP), whereas locomotor activities were measured by an infrared light beam crossing system as described below. After a baseline measurement for 5 min, an anesthetic agent was acutely delivered, and locomotor activity was recorded for 10 more minutes.

### Behavioral and locomotor measurements using an activity monitoring system

The mice’s spontaneous locomotor activity during the continuous CSF collection or intrathecal drug delivery experiment was monitored in an open-filed cage (43 × 43 cm) with high-density arranged infrared sensors (Scanet, MV-40, Melquest, Toyama, Japan) [21, 22]. The infrared sensors were distributed in all directions parallel to the floor (37.5 mm from the floor). The mice were allowed to move freely on the floor, with their movements detected by infrared sensors and recorded every 0.1 s.

### Statistical analysis

All data are expressed as the mean ± standard error of the mean. Two-group comparisons were performed by an unpaired t-test, unless otherwise stated. Comparisons among three or more groups were performed by analysis of variance and the Tukey–Kramer test, unless otherwise stated. p values < 0.05 were considered statistically significant.

## Results

### Chronic dural port for continuous collection of CSF in a free-moving mouse

**Fig. 1:**
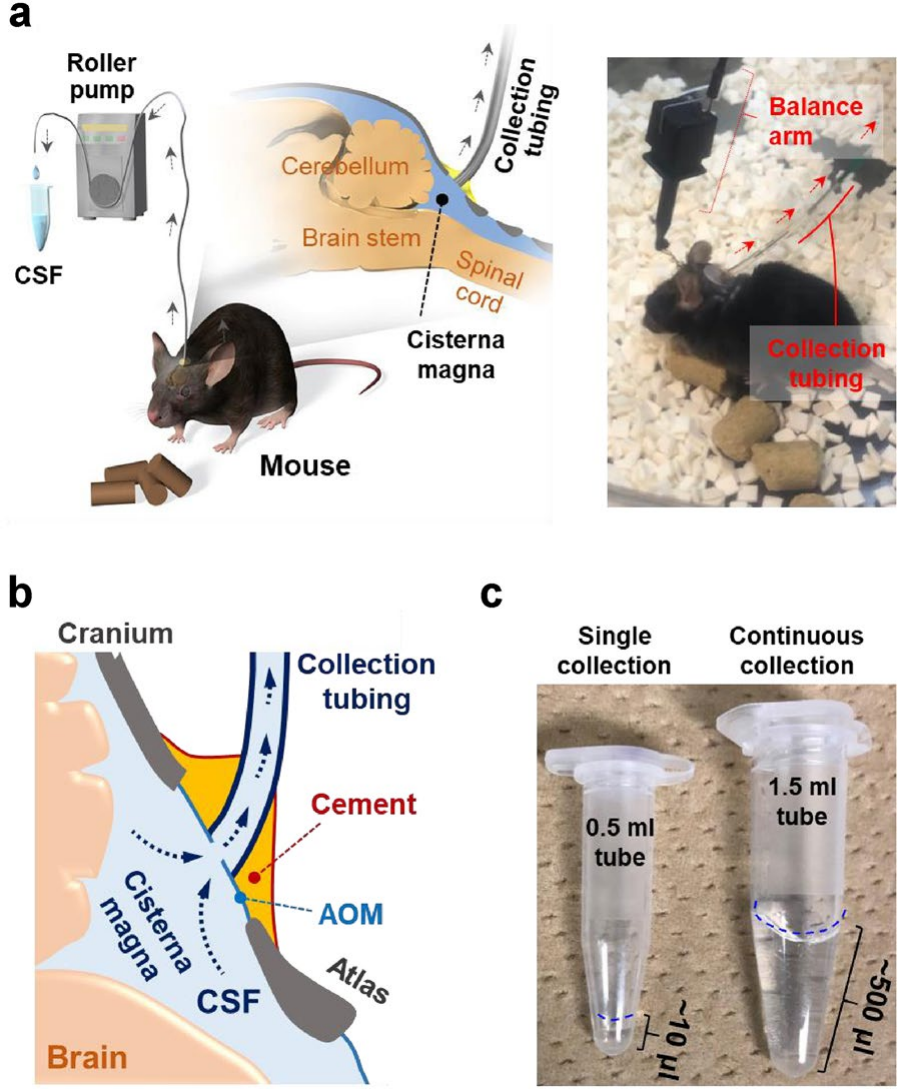
Continuous collection of a large volume of CSF in the free-moving mouse. **a** Schematic of the continuous CSF collection in the free-moving mice (left panel). Mouse in the movement-response rotating cage (right panel). Mouse is connected to the sensor-integrated balance arm to suspend and prevent twisting of the CSF tubing. **b** CSF tubing is fixed onto the AOM with dental cement covering the caudal end of the cranium, CSF tubing, and rostral end of the atlas. CSF is collected via a small hole (~ 0.2 mm of diameter, needs to be smaller than the inner diameter of the tubing) introduced in the AOM and through the CSF tubing connected to the pull continuous flow roller pump (2–5 µL/h). Note that the end of the CSF tubing does not enter the subarachnoid space. **c** A large volume of clear CSF collected from a single mouse during five consecutive days (right). Conventional single-collection method yields approximately 10 µL of CSF per mouse (left)

Figure 1 shows the schematic for continuous CSF collection using the CDP method in an awake, free-moving mouse. The CDP is installed on the CM at the dorsal part of the mouse’s neck. The CSF collection tubing from the CDP is connected to a roller pump (or syringe pump) to withdraw CSF at a constant flow rate (Fig. 1a, left). This method does not require anesthesia for the mouse during an experiment, thereby enabling continuous long-term collection of CSF under ad libitum conditions for water and food intake. The mouse is tethered with the sensor-integrated balance arm and placed into the movement-response caging system [18] designed to allow unrestricted movement of the animal without applying pressure to the probe assembly (Fig. 1a, right, Additional file 1: Figure S1).

**Fig. 2:**
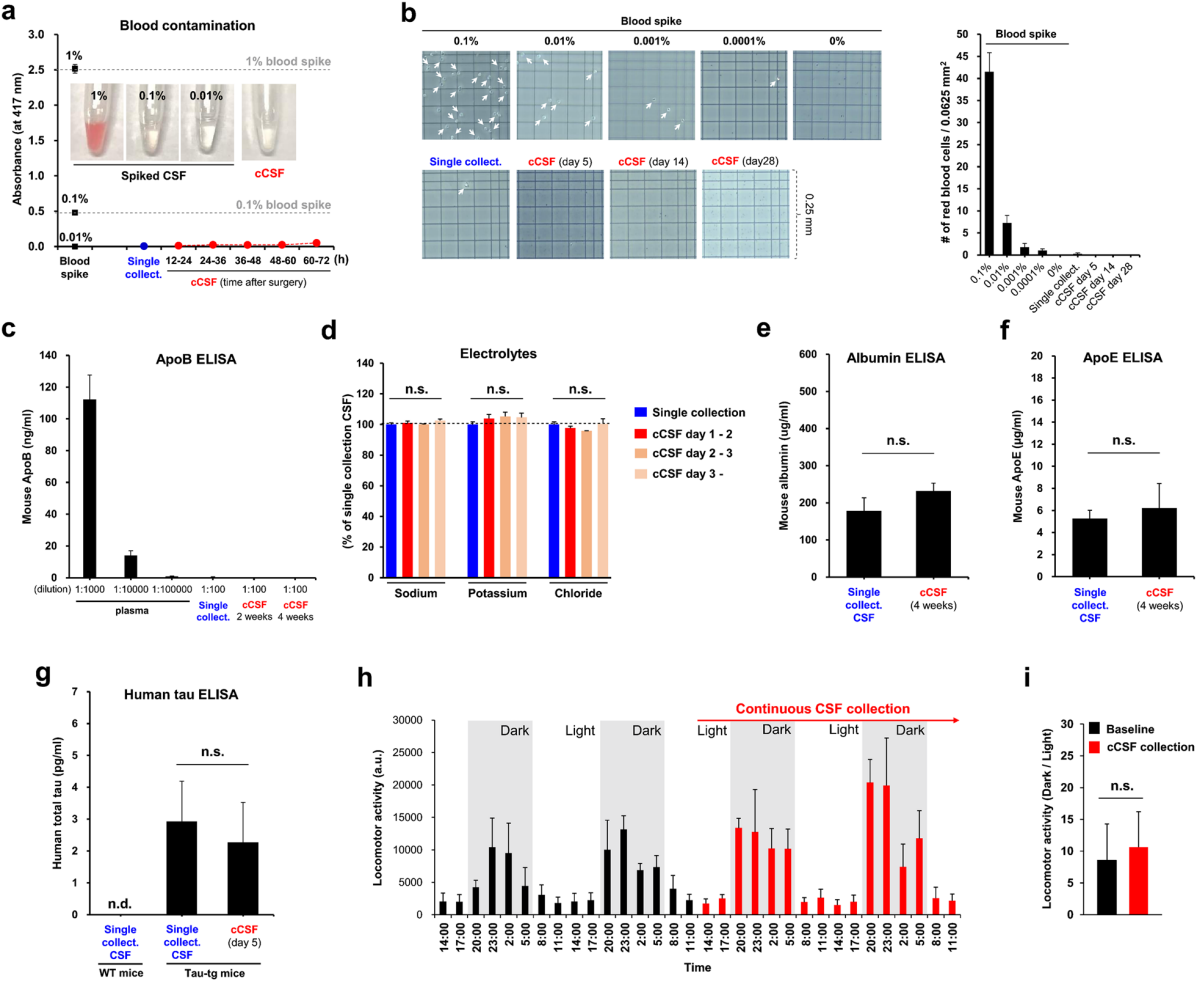
Continuous collection of high-quality CSF from naturally-behaving mice. **a** Quantitative assessment of CSF blood contamination using a highly sensitive spectrometer method. Hemoglobin contamination levels (absorbance at 417 nm) from blood-spiked CSF (intentionally spiked with 1%, 0.1%, or 0.01% vol/vol of blood, black), mouse CSF collected by the conventional single collection technique (blue), and cCSF at various postoperative times (red). Insets are representative images of spiked CSF and cCSF. **b** Direct blood count under the phase contrast microscope for sensitive detection of CSF blood contamination. (b, left) Representative images of red blood cell (RBC) contamination in the blood-spiked CSF, single collection CSF, and cCSF. The arrows indicate RBCs. (b, right) The RBC count in the CSF samples (n = 3–4). **c** ELISA measurement of plasma abundant protein ApoB in the single collection CSF, cCSF, and plasma. Plasma samples were diluted to 1:1,000, 1:10,000, 1:100,000; CSF samples were diluted to 1:100 for the measurements. **d** Normal cCSF electrolyte concentrations during long-term continuous collection. Sodium, potassium, and chloride levels measured in the single-collection CSF and cCSF at various postoperative times (n = 4–6, one-way ANOVA). **e**, **f**) Quantifications of albumin (**e**) and ApoE protein (**f**) in the single collection CSF and cCSF (n = 4–10). **g** Quantification of human total tau levels in the single collection CSF and cCSF. Seven-month-old tau-transgenic PS19 mice and wild-type littermates were used for the experiment (n = 3–7). **h** Mice’s normal locomotor activities and circadian rhythms during long-term cCSF collection. The locomotor activities were measured every 3 h before (baseline, black) and during cCSF (red) under the normal 12:12 h light-dark cycle. **i** Locomotor activity dark/light ratio during baseline and cCSF is shown (n = 3, Student’s t-test). * CSF* cerebrospinal fluid, *AOM* atlanto-occipital membrane, *i.d.* inner diameter, *cCSF* CSF obtained by the continuous collection method, *n.d.* not detected, *n.s.* not significant

The detailed structure of the CDP is shown in Fig. 1b. A small hole is made on the AOM covering the CM and connected to the CSF collection tubing. The AOM hole is made slightly smaller (~ 0.2 mm) than the internal diameter of the CSF collection tubing (0.3 mm) to prevent leakage of the CSF during collection. The CSF is collected by applying negative pressure to the CSF collection tubing with a roller (or syringe) pump. Dental cement is used to fix the CSF collection tubing, AOM, and surrounding bone structures, including the occipital crest and the anterosuperior border of the atlas, thereby enabling the stable collection of CSF without leakage in the free-moving mouse. Notably, the tip of the CSF collection tubing is not inserted into the intrathecal space; it is instead attached and fixed onto the AOM covering the small hole (Fig. 1b). This is an important feature of the CDP method because it prevents tissue damage during long-term continuous CSF collection in free-moving mice (Fig. 2).

In contrast to a single-collection method [11, 23], the CDP method allows for the collection of a large volume of CSF from a single mouse (Fig. 1c, left). Continuous CSF collection via the CDP enables the collection of approximately 500-µL CSF from a single mouse over a 5-day collection period when the flow rate is maintained at 0.067 µL/min (Fig. 1c, right). This sampling flow rate is much lower than the physiological production rate (~ 0.35 µL/min) of mouse CSF [1, 24, 25]. Continuous CSF collection via the CDP can be conducted in a free-moving mouse given ad libitum access to water, which can maintain its physiological condition because the rate of CSF sampling is sufficiently lower than the physiological production rate.

### Continuous collection of high-quality CSF from naturally behaving mice

We also investigated the quality of the CSF collected via the CDP. Blood contamination in the CSF can have a significant impact on the analytic outcomes in biomarker research [26]. A high-sensitivity spectrophotometry method [10] was used to measure blood contamination in the collected CSF. This method detected blood contamination down to 0.001%, a level undetectable by eye. As shown in the inset photos in Fig. 2a, contamination with 0.1–1% blood was visible, whereas blood contamination lower than 0.01% was undetectable to the naked eye. Continuously collected CSF (cCSF) via the CDP had a clear appearance, and blood contamination was undetectable by the high-sensitivity spectrophotometer method, indicating that the collected CSF was as clean as the CSF collected via a conventional single-collection technique (Fig. 2a). Notably, no significant blood contamination was detected in the cCSF during long-term collection over a few days. Using two other sensitive methods, we further confirmed that the cCSF was not contaminated with blood. We performed a direct blood cell count under a phase contrast microscope, which is more sensitive for blood contamination than the spectrophotometer method and confirmed that the CSF was free from red blood cells (Fig. 2b). We also measured the amount of apolipoprotein B (ApoB), an abundant plasma protein in the cCSF, using a sensitive ELISA (Fig. 2c). The ApoB concentration in the cCSF was 1000-fold lower than that in the plasma, which was under the detection level of the ELISA. No notable blood contamination was detected in the cCSF even after long-term collection (e.g., four weeks) (Fig. 2b, c). We also measured the levels of basic CSF electrolytes, such as sodium, potassium, and chloride, in the CSF collected using a conventional single-collection method and the CDP method (cCSF). The cCSF concentrations of these electrolytes were maintained within the physiological range [27] over the three-day collection and were comparable to those determined in CSF obtained by a single collection (Fig. 2d). To further validate the quality and nature of the cCSF, we quantified the amount of proteins that are known to be detectable in normal CSF, such as albumin and ApoE [28]. Albumin is the major protein found in CSF and its concentrations in the single-collection CSF and cCSF (at 4 weeks) were within the physiological range (~ 200 µg/mL), with no significant difference between them (Fig. 2e). ApoE is one of the main brain-derived proteins in CSF, with an intrathecal synthesis of 90% and a physiological concentration of approximately 6 µg/mL. The ApoE concentrations in the single-collection CSF and cCSF (at 4 weeks) were comparable, and both were within the physiological range (Fig. 2f). These findings provide additional validation that cCSF is not blood contaminated and can be used as a valid resource for biomarker research. Notably, long-term (four-week) collection did not appear to affect the concentrations of these CSF proteins (Fig. 2e, f).

We also measured tau protein concentrations in the CSF samples of tau-transgenic PS19 and wild-type mice (Fig. 2g). Tau is a well-validated neurodegenerative marker protein for Alzheimer’s disease and other related neurodegenerative disorders. In this study, we used a human-tau-specific ELISA to detect pathological tau in the CSF of tau-transgenic PS19 mice that overexpress human mutant tau. High tau concentrations were detected in the single-collection CSF and cCSF of the tau-transgenic mice (Fig. 2g), reflecting brain neurodegeneration. There was no significant difference in CSF tau levels between the single-collection CSF and cCSF (day 5), suggesting that the CDP method can be used for monitoring the CSF concentrations of neurodegenerative markers.

We also investigated the impact of continuous CSF collection via the CDP on mouse behavior. The locomotor activity of the animal was quantitatively measured using an infrared beam-based activity detection system [29]. Baseline measurements were obtained for two days, and then the continuous CSF collection was started in the middle of the light phase of the second day (12:00 noon on day 2) and continued for two more days (Fig. 2 h). The mice under continuous CSF collection maintained a normal circadian rhythm, with no significant differences in the dark/light ratio of locomotor activity between baseline and during the cCSF collection (Fig. 2i), suggesting that continuous CSF collection via CDP has no impact on their physiological behavior.

### Minimal tissue damage on brain and spinal cord after long-term CSF collection via CDP

CSF collection via conventional CM cannulation (CMc) can cause tissue damage after long-term experiments lasting several days or weeks, especially when using small-sized rodents, such as mice.

**Fig. 3:**
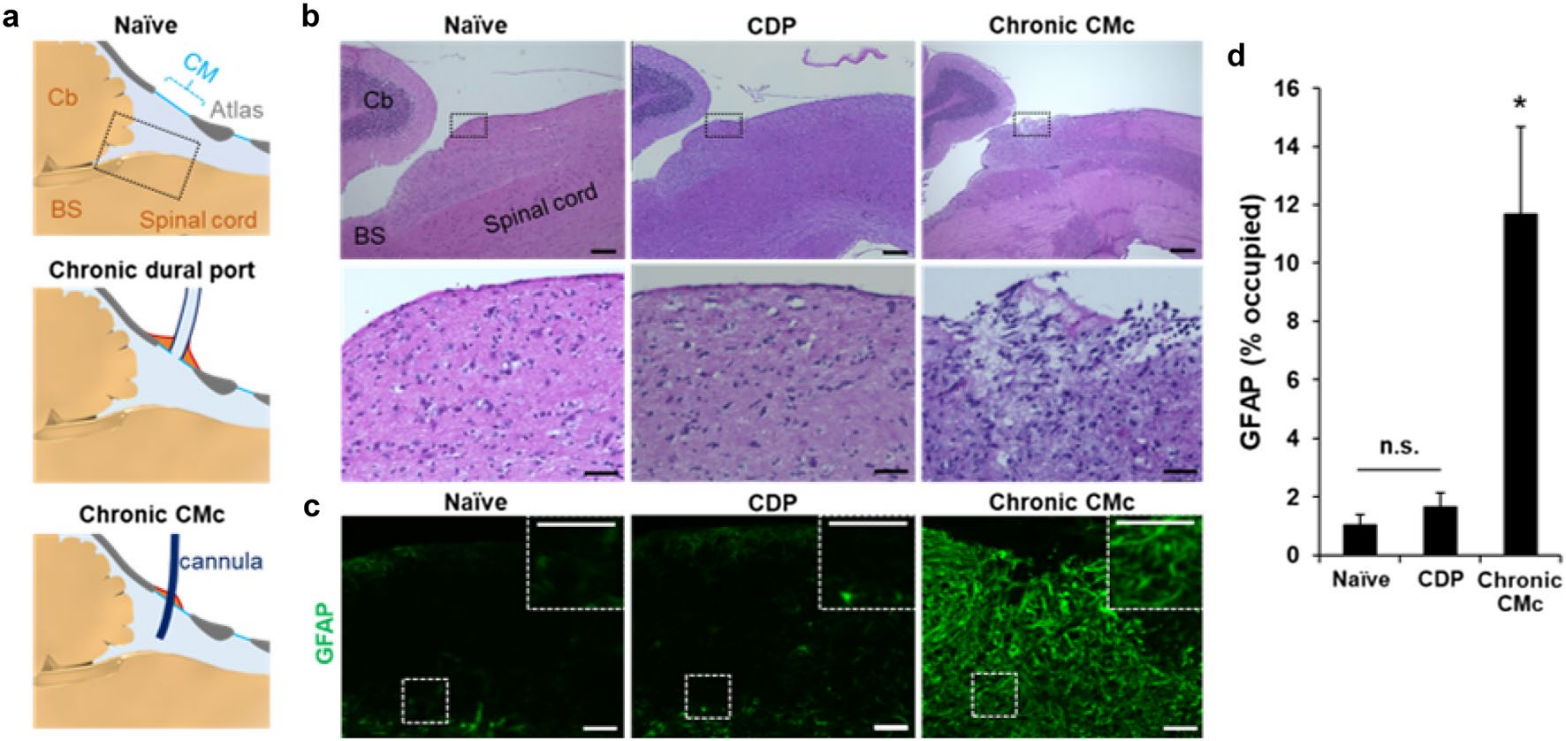
Minimal histological damage to brain and spinal cord after chronic dural port implantation **a** Scheme of the sagittal view of the mouse brain and spinal cord area adjacent to the CM of intact and sham-operated mice (top), CDP (middle), and chronic CMc (bottom). Dotted square indicates the areas where the histological appearances are shown in **b**, **c**. **b**, **c** Histological integrity and astrocyte reactivity two weeks after CDP or chronic CMc implantation compared to the sham-operated mice. (**b**, top panels) H&E-stained sagittal sections of the BS and spinal cord area adjacent to CM of sham-operated mice, CDP, and chronic CMc. Scale bar, 200 μm. (**b**, bottom panels) High magnification of the H&E-stained sagittal sections corresponding to the dotted outlined area in the top panels. Scale bar, 50 μm. **c** GFAP-stained sagittal sections corresponding to the outlined area in the top panels of (**b**); high magnification of GFAP-positive cells in the areas outlined with white dotted square are shown in insets. Scale bar, 50 μm. **d** Quantification of GFAP-positive cells in the area corresponding to the dotted outlined area in the top panels of (**b**). n = 6/group. *p < 0.05 versus naïve and CDP mice. One-way ANOVA and a subsequent Tukey–Kramer test were performed. Data presented are the mean ± SEM. * BS* brain stem, *Cb* cerebellum, *CDP* chronic dural port, *CM* cisterna magna, *CMc* cisterna magna cannulation, *H&E* hematoxylin-eosin, *GFAP* glial fibrillary acidic protein

In contrast to the conventional CMc method, the CDP does not require the intrathecal insertion of a cannula for CSF collection (Fig. 3a); therefore, it is expected to reduce tissue damage. Figure 3b–d shows a sagittal section of the brain and spinal cord seven days after the surgery for CDP installation. There was no apparent tissue injury or inflammation with the CDP method, whereas the CMc method showed a clearly visible injury on the surface of the brain stem (Fig. 3b), an injury that was accompanied by an excessive accumulation of GFAP-positive astrocytes (Fig. 3c). The number of GFAP-positive astrocytes in the brain tissue underneath the CM was significantly higher with the CMc method, whereas the mice subjected to the CDP method had comparable GFAP-positive astrocyte numbers to those of naïve mice (Fig. 3d). These results indicate that the CDP method provides long-term CSF collection from mice with minimal tissue damage.

### Application of the CDP method for continuous real-time glucose monitoring in the CSF

**Fig. 4:**
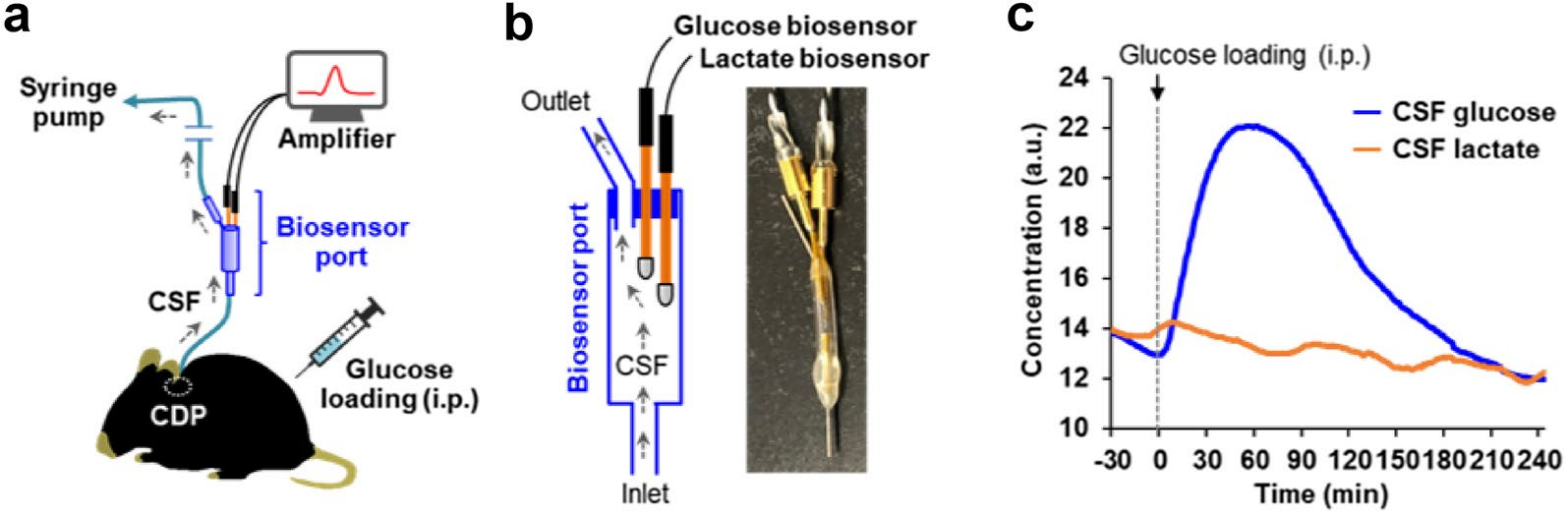
Monitoring of real-time changes in CSF glucose concentration in the free-moving mice. **a** Schematic of continuous CSF collection via CDP for monitoring glucose and lactate levels during glucose loading. CSF levels of glucose and lactate were continuously and quantitatively measured using biosensors attached to the middle of the collection tubing (biosensor port). CSF was collected at the flow rate of 0.05 µL/min using a syringe pump. Mice were intraperitoneally injected glucose (2 mg/g body weight) during continuous CSF collection. **b** Schematic of biosensor port. Two electrodes of the glucose and lactate biosensors are installed within the port. Electrodes are exposed to the continuous flow of CSF and provide real-time changes in glucose and lactate concentrations. **c** CSF levels of glucose (blue line) and lactate (orange line) after intraperitoneal glucose loading. A representative data is shown from a wild-type mouse. * CDP* chronic dural port, *CSF* cerebrospinal fluid, *i.p.* intraperitoneal injection

We applied the CDP method to monitor the dynamic metabolism of glucose and lactate in the CSF during peripheral glucose loading. To this end, we employed enzyme-based biosensors, which enable second-by-second recording of each molecule’s change in concentration (Fig. 4). The biosensors for glucose and lactate were attached to the middle of the CSF collection tubing (biosensor port); each biosensor electrode was connected to the recording software with an amplifier (Fig. 4a) [19]. The mouse was housed in a movement-response caging system, which allows for free movement during the experiment. Figure 4b shows the structure of the biosensor port in which electrodes are exposed to the continuous flow of CSF to monitor real-time changes in glucose and lactate concentrations. The inlet of the port is connected to the CDP, and the outlet is connected to a syringe pump to withdraw CSF at a constant flow rate.

The mouse was administered an intraperitoneal injection of glucose during the continuous CSF collection at a flow rate of 0.05 µL/min. The CSF glucose concentration showed a rapid increase after intraperitoneal glucose loading, reaching its peak at approximately 50 min, followed by a gradual decrease down to baseline level at approximately 180 min (Fig. 4c). The CSF lactate concentration showed no significant change during the glucose loading (Fig. 4c), indicating the specificity of each biosensor for each molecule. The CDP method for continuous CSF collection, combined with the glucose/lactate biosensors, allows for the real-time monitoring of dynamic glucometabolic changes in the CNS at a one-second temporal resolution in free-moving mice.

### Injection of a CSF tracer via the CDP and its temporal and spatial distributions in mice

**Fig. 5:**
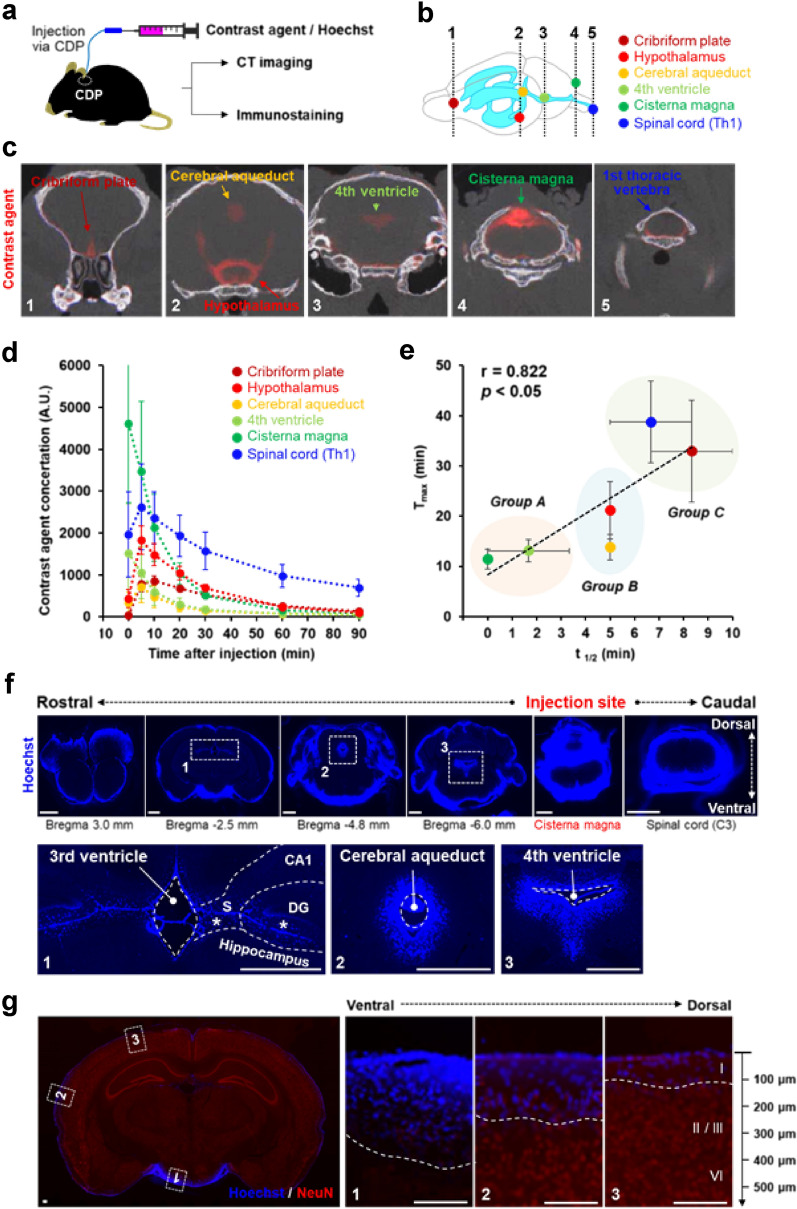
Injection of CSF tracer via CDP and its temporal and spatial distributions in mice. **a** Schematic of acute injection of tracers into the CSF system of free-moving mice via CDP and subsequent analysis of the tracers’ dynamic distribution and metabolism by micro-CT imaging and brain sections. **b** Schematic of mouse brain ventricles. **c** Representative coronal brain images of micro-CT with contrast agent (shown in red). Numbers in the bottom right corner of each image correspond to the levels of the sections indicated in (**b**). **d** Quantification of the contrast agent concentrations in each region. Radiodensity values of the contrast agent were sequentially measured at each time point. n = 3/group. Data are presented as the mean ± SEM. **e** Relationship between half-life (t1/2) and Tmax values of the contrast agents in each region. Each plot represents the mean of three animals with SEM error bars. p < 0.05, Spearman rank test. Subregions were divided into three groups depending on the metabolic rates of tracers. Group A; regions with rapid distribution and fast clearance of the tracer; Group B; regions with intermediate metabolism of the tracer, Group C; regions with delayed distribution with slow clearance of the tracer. **f** Representative images of coronal sections of mouse brain after an acute injection of Hoechst solution into the CM (injection site) via CDP. (f, bottom panels) High magnification of the sections corresponding to the dotted outlined area in the top panels. Scale bar, 1,000 μm. **g** Representative image of the coronal section immunostained with neuronal marker NeuN. High magnifications of the areas corresponding to the dotted outlined area in the right panel are shown in the left three panels. Cortical layers are indicated by Roman numerals in panel 3. Scale bar, 200 μm. *CDP* chronic dural port, *CT* computed tomography, *DG* dentate gyrus, *S* subiculum, *Tmax* time to peak concentration

The CDP can also be used as an injection port for various compounds, such as CSF tracers and drugs. We investigated the spatio-temporal distribution of CSF tracers injected into the intrathecal space via the CDP (Fig. 5). We injected a contrast agent used for microcomputed tomography (micro-CT) imaging via the CDP, followed by sequential CT imaging of the mouse brain for 90 min (Fig. 5a–e). CT images of five coronal sections from different parts of the mouse brain (cribriform plate, hypothalamus, cerebral aqueduct, fourth ventricle, CM, and spinal cord) (Fig. 5b) were used to quantitatively measure the concentrations of the injected contrast agent (Fig. 5c). The contrast agent injected via the CDP showed a quick and broad rostral-to-caudal distribution from the cribriform plate to the spinal cord sections (Fig. 5c).

Moreover, we evaluated the dynamic metabolism of the injected tracer in each part of the intrathecal space by measuring the temporal changes in the concentrations and calculating the half-life (t1/2) and the time to peak concentration (Tmax) values (Fig. 5d, e). In the areas adjacent to the infusion site (CDP), such as the CM and the fourth ventricle, the injected tracer immediately reached its peak concentration (Tmax, ~ 10 min), followed by a rapid decrease to the baseline levels by 60 min (t1/2, < 3 min) (Group A in Fig. 5e). In the hypothalamus and cerebral aqueduct, the injected tracer reached its peak a few minutes later (Tmax, ~ 10–20 min), followed by a gradual decrease to the baseline levels by 60 min (t1/2, ~ 5 min) (Group B in Fig. 5e). In the areas distant from the infusion site, such as the cribriform plate and spinal cord, the injected tracer took longer to reach its peak (Tmax, > 30 min) and to wash out (t1/2, 7–8 min) (Group C in Fig. 5e).

We also histologically assessed the spatial distribution and degree of penetration of a tracer injected into the brain parenchyma via the CDP (Fig. 5f, g). We injected a single dose of a water-soluble nuclear-staining dye (Hoechst) via the CDP, harvested the brain at 60 min, and examined the nuclear staining in brain sections taken from different areas (Fig. 5f). A broad distribution of the injected dye was noted, ranging from the most rostral part of the olfactory bulb (at the Bregma 3.0 mm) to the most caudal part of the spinal cord (at the cervical vertebra C3), with the highest staining intensity at the injection site of the CM (Fig. 5f, top panels). Notably, the dye injected retrograde into the CM passed to the fourth ventricle (Fig. 5f, indicated as a dotted rectangle 3), through the cerebral aqueduct (Fig. 5f, indicated as a dotted rectangle 2), and then to the third ventricle (Fig. 5f, indicated as a dotted rectangle 1). Nuclear staining was observed in the cells of the hippocampal dentate gyrus and subiculum (Fig. 5f, lower panel (1)), indicating that these brain areas can be exposed to a drug injected via the CDP into the CM. Figure 5 g shows a coronal section at the dorsal hippocampus (Bregma − 2.5 mm) after immunostaining with the neuronal cell marker NeuN. The nuclear staining was stronger in the ventral part of the brain than in any other region when the dye was injected into the CM with the mouse placed in the normal prone position (Fig. 5 g, indicated as a dotted rectangle 1), suggesting that an intrathecally injected tracer tends to be retained in the basal part of the brain. Deep penetration of the tracer into the brain parenchyma was observed at the ventral part of the brain, extending to approximately 400 μm from the surface (Fig. 5 g, indicated as dotted rectangle 1). The lateral (~ 300 μm deep) and dorsal (~ 100 μm deep) parts of the brain showed a more superficial penetration of the tracer (Fig. 5 g, indicated as dotted rectangles 2 and 3, respectively).

### Application of the CDP method for the intrathecal administration of drugs and real-time behavioral assessment

**Fig. 6:**
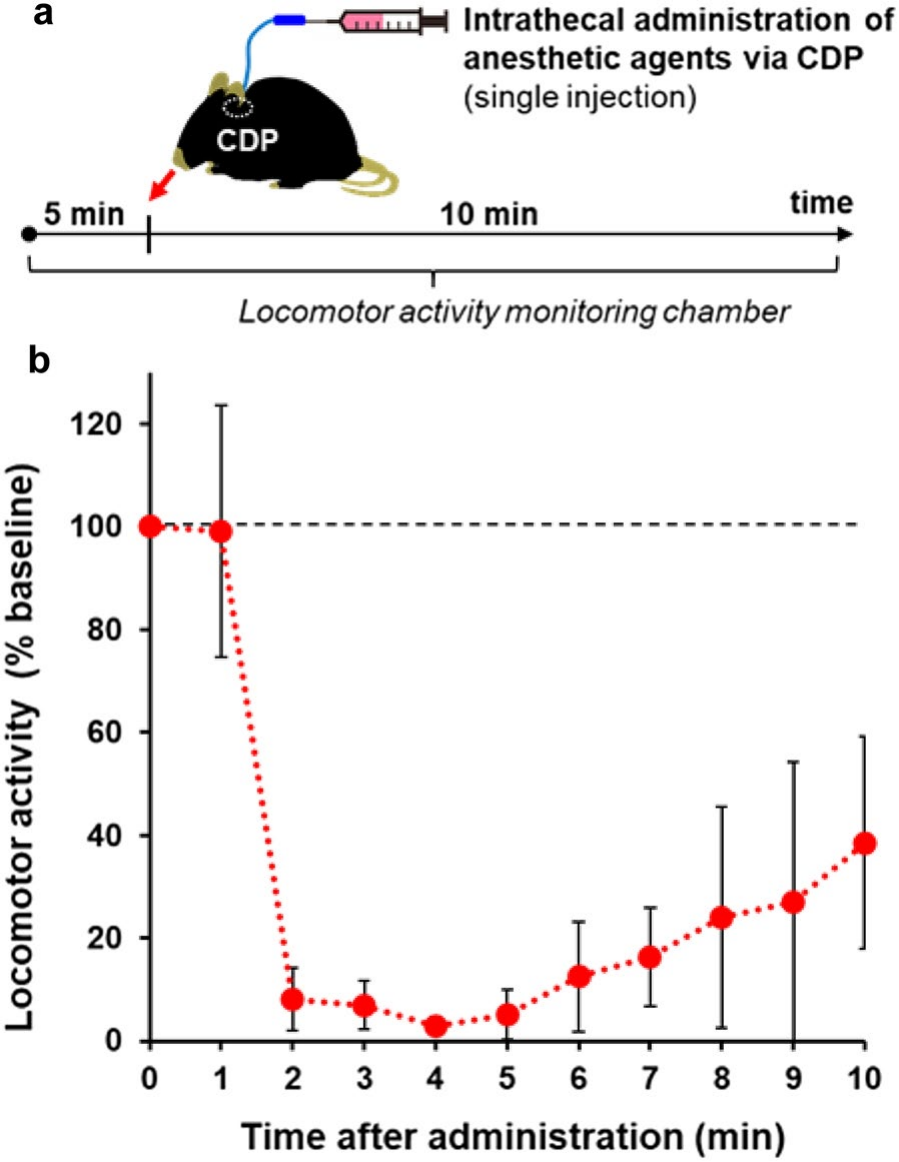
Acute intrathecal administration of drug via CDP and behavioral assessment in the free-moving mouse. **a** Time course of the experiment. Anesthetic agent (midazolam) was acutely injected via CDP in the free-moving mice (5 µL in 1 s through a syringe connected to the CDP), while locomotor activities were quantitatively measured by the infrared light beam crossing system. **b** Locomotor activity after the intrathecal administration of anesthetic agent via CDP in the free-moving mice. n = 4/group. Data are presented as the mean ± SEM. * CDP* chronic dural port

The CDP method enables the intrathecal administration of centrally acting drugs and the real-time assessment of behavioral changes in awake, free-moving mice. We injected a single acute dose of anesthetic agent via the CDP while quantitatively measuring the animal’s locomotor activity with high-temporal resolution using an infrared light beam crossing system (Fig. 6a). The mice showed rapid decreases in locomotor activity immediately after the anesthetic agent was administered (within 2 min), suggesting a direct pharmacological action of the intrathecally administered drug. The animals’ locomotor activity started to increase at 6 min after drug administration, indicating a relatively fast recovery from the anesthetic to the awake state (Fig. 6b).

## Discussion

Brain tissue is surrounded and protected by a bony structure, which makes it difficult to perform biopsies and the direct delivery of therapeutic drugs. CSF collection and intrathecal drug administration have therefore been employed as alternative methods. The small size of the intrathecal space in mice impedes the stable collection of a large volume of CSF for detailed analysis of its components, which has been the main bottleneck for CSF biomarker research using mouse models. In this study, we developed a CDP method as a novel approach for CSF collection that allows for continuous long-term CSF sampling in awake, free-moving mice. This method enables the collection of large volumes of high-quality CSF suitable for biomarker research and can also be used for the intrathecal delivery of drugs for the real-time monitoring of their pharmacological effect on the behavior of free-moving mice.

The volume of CSF obtained through conventional methods, such as CM puncture (~ 10 µL per single mouse) [9, 10], is insufficient for measuring multiple biomarkers, even when using a highly sensitive ELISA, and requires the pooling of CSF from multiple animals to obtain a large enough volume for measurement. The CDP method enables the continuous long-term collection of CSF from a single mouse, thereby obtaining a sufficiently large volume of CSF for the comprehensive profiling of CSF components. Sampling can be performed under ad libitum conditions for water and food intake, which prevents dehydration during continuous long-term CSF collection. The flow rate of the CSF collection used in this study (< 0.07 µL/min) was lower than the physiological production rate of mouse CSF (~ 0.35 µL/min) [1, 24, 25]. We confirmed that long-term CSF collection at this flow rate does not have a significant impact on CSF electrolyte concentrations (Fig. 2b) and the physiological behavior of mice, such as their diurnal circadian rhythm (Fig. 2 h, i). Given that CDP enables the collection of large volumes and repeated serial collection from a single mouse, it can be useful for monitoring long-term changes in biomarker levels and the efficacy of therapeutic intervention over time, which can reduce the number of animals needed for an experiment. The continuous collection of a large volume of CSF from a single mouse could provide advantages for the discovery of biomarkers; however, the possibility cannot be ruled out that the continuous collection of CSF might have a potential impact on the normal CSF turnover and form errors in interpreting the obtained results. Although we confirmed that the CSF collected via a single CM puncture and the cCSF collected via the CDP method had comparable levels of physiological and disease-related proteins, such as albumin, ApoE, and tau (Fig. 2e–g), the potential impact of the continuous large volume collection on CSF turnover and metabolism should be taken into consideration. Microdialysis is another method for collecting CSF in a continuous manner [5]. The size-exclusion nature of the microdialysis membrane, which restricts the size of molecules collected in the dialysate, is the main drawback of using microdialysis for this purpose. High-molecular-weight proteins cannot be collected by microdialysis. The risk of tissue damage from the insertion of the microdialysis probe into the intrathecal space is another limitation of microdialysis for CSF collection. With the CDP method, whole CSF components can be collected with minimal tissue injury.

The potential impact of protein degradation and any biochemical alteration in the cCSF samples due to long-term exposure to room temperature during continuous collection should also be considered. In our experimental setting, it took approximately 8 h for the cCSF samples traveling through the collection tubing to reach the refrigerated fraction collector. Our preliminary experiments confirmed that the values for the CNS-related protein markers, such as tau, phosphorylated tau, α-synuclein, neuron-specific enolase, and GFAP did not significantly change after a 24-h exposure to room temperature (data not shown). In fact, the levels of CSF markers, such as tau, ApoE, and albumin, were not significantly different between the single CM puncture and the long-term collection via CDP (Fig. 2e–g), suggesting that the exposure to room temperature within the collection tubing had a minimal impact on the values of these protein markers. In addition, nonspecific adsorption to tubing materials should be considered when measuring the concentrations of any proteins or drugs of interest and should be initially examined as needed.

The quality of the collected CSF is critical, especially for biomarker development research [10, 26]. Blood contamination in CSF significantly affects biomarker analysis, especially when the molecules’ concentration (such as alpha-synuclein) is supposed to be higher in blood than in CSF [26]. Blood contamination can also increase nonspecific noise reactions due to a large number of hydrophobic immunoglobulin components, resulting in sample-related biases [30]. Tissue injury during CSF collection and intrathecal drug delivery, especially when using the intrathecal cannulation method, can increase the concentrations of neurodegenerative markers, such as tau and neurofilament, as artifacts. Notably, the CDP method does not require that the CSF collection tubing be inserted into the intrathecal space and therefore significantly reduces the likelihood of tissue injury, even in long-term experiments. The large volume of CSF collected via CDP was free of detectable blood contamination (< 0.0001%) (Fig. 2a–c) and caused minimal tissue damage and accumulation of inflammatory cells (Fig. 3).

The main mechanisms of CSF production, circulation, and absorption, as well as the dynamic metabolism of CSF components, including proteins, electrolytes, and neurochemicals, are still largely unknown [31]. Knowledge of these fundamental aspects of CSF metabolism is essential for developing biomarkers and understanding the pathogenesis of CNS disorders related to CSF dysregulation, such as normal pressure hydrocephalus [32]. The continuous collection of CSF via CDP combined with specific and sensitive biosensors enables the real-time measurement of dynamic changes in the CSF components of free-moving mice (Fig. 4), which provides a useful platform for basic research on CSF metabolism. To the best of our knowledge, this is the first report to demonstrate the monitoring of dynamic changes in CSF glucose levels at a one-second temporal resolution in free-moving mice (Fig. 4c). The sensitive measurement of CSF glucose and other neurochemicals at a high-temporal resolution can be a valuable tool for understanding the pathophysiological changes in the glucometabolic state in CNS disorders [33, 34].

The CDP method can also be applied to intrathecal drug delivery for therapeutic development. Intrathecal administration is employed for treating several CNS disorders, such as pain, multiple sclerosis, and brain tumors [35, 36], because this approach can bypass the blood–brain and blood–CSF barrier and enable direct pharmacological action on the CNS. In this report, we demonstrated that the CDP method combined with a behavioral assessment tool, such as an activity cage for recording spontaneous coordinate activity, enables the real-time evaluation of a centrally acting drug delivered into the intrathecal space in free-moving mice (Fig. 6). Intrathecal drug delivery via CDP in free-moving mice with minimal physical restraint has key advantages over conventional methods, such as intrathecal canulation [13, 14] and direct injection into the lateral ventricle [15], in which tissue injury-associated motor and cognitive impairment can be a serious problem in behavioral assessment.

Using the CDP method, we demonstrated the spatio-temporal distribution and metabolism of an intrathecally delivered CNS tracer (Fig. 5), which was injected into the CM, was widely distributed in the intrathecal space; notably, reached the hippocampal area (Fig. 5f). However, there was a significant difference in the kinetic parameters, such as t1/2 and Tmax, of the brain regions (Fig. 5d, e), suggesting the complexity of CSF circulation and metabolism, which should be taken into consideration when evaluating the therapeutic effects of intrathecally administered drugs. The kinetics of tracer administration, such as the amount and speed of injection, could affect the kinetic pattern differences among brain areas.

Furthermore, the degree to which the injected compound penetrated the parenchyma varied among brain areas (Fig. 5g). Multiple factors, such as brain area, size, and the circulation of CSF surrounding the brain tissue, can influence drug penetration. Moreover, the expression and activity levels of certain transporters within specific brain areas could be related to the distribution and penetration of injected tracers and drugs. All of these factors could affect the intrathecal administration’s efficacy.

The drawbacks of the CDP method include (1) the potential limitation of neck movement due to CDP fixation with dental cement and (2) the requirement for surgical training. The CDP method requires fixation of the CSF collection tubing to the AOM and surrounding bone structures, including the occipital crest and anterosuperior border of the atlas, which is important for stable CSF collection in free-moving mice. We observed no apparent behavioral alterations after the CDP installation. Mice with CDP showed rapid recovery from the surgery, and surgery-related body weight loss was restored within four days after the operation (data not shown). Notably, the mice with the CDP implant survived as long as a few months after surgery, indicating the minimal physical stress exerted using this method on animals. The study mice maintained a normal circadian rhythm, even during continuous CSF collection (Fig. 2c, d). Although this surgical procedure requires training, a basic animal surgery setup is sufficient, and the step-by-step instructions described in the Methods section should be helpful for researchers to master the technique. In our experiment, the animals’ mortality rate after the CDP surgery was less than 2% (data not shown).

## Conclusions

The CDP method provides a unique and valuable platform for CSF biomarker research and therapeutic development using animal models relevant to CNS disorders. An implanted CDP allows for the repeated and long-term access to the CSF and intrathecal space in awake, free-moving mice. A large volume of high-quality CSF collected from mice under physiological conditions can be useful for identifying novel relevant biomarkers.

## Supplementary Information

**Additional file 1: Figure S1**. Schematic of continuous CSF collection in the free-moving mice in the movement-response rotating cage. The mouse is connected to the sensor-integrated balance arm via a steel wire anchor attached on the skull.

## Data Availability

The datasets used and/or analyzed during the current study are available from the corresponding author on reasonable request.

## Abbreviations

AOM: atlanto-occipital membrane
CDP: chronic dural port
CM: cisterna magna
CNS: central nervous system
CSF: cerebrospinal fluid
cCSF: continuously collected CSF
CT: computed tomography
NPH: normal pressure hydrocephalus
Tmax: time to peak concentration
