# Supplementary material for: A novel chronic dural port platform for continuous collection of cerebrospinal fluid and intrathecal drug delivery in free-moving mice
Source: Fluids Barriers CNS. 2022 May 3;19:31. doi: 10.1186/s12987-022-00331-1 (PMC9066940; doi:10.1186/s12987-022-00331-1)
Supplement: Supplementary file 1 — Additional file 1: Figure S1. Schematic of continuous CSF collection in the free-moving mice in the movement-response rotating cage. The mouse is connected to the sensor-integrated balance arm via a steel wire anchor attached on the skull. [file 12987_2022_331_MOESM1_ESM.docx]

**Supplemental Figure 1**

**
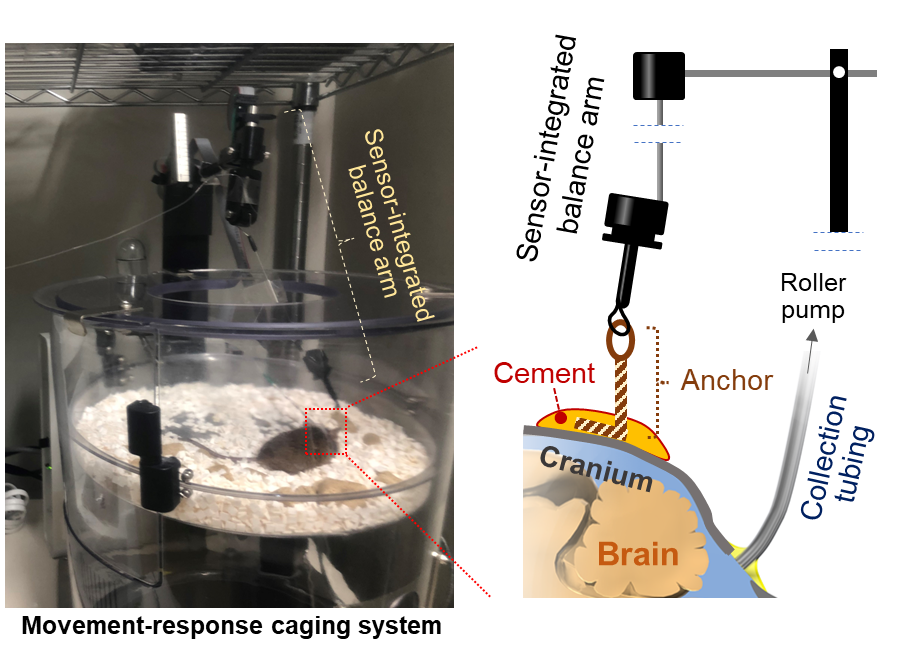
**

Schematic of continuous CSF collection in the free-moving mice in the movement-response rotating cage. The mouse is connected to the sensor-integrated balance arm via a steel wire anchor attached on the skull.
